# 6‐Shogaol induces apoptosis in acute lymphoblastic leukaemia cells by targeting p53 signalling pathway and generation of reactive oxygen species

**DOI:** 10.1111/jcmm.16528

**Published:** 2021-05-03

**Authors:** Somayeh Najafi Dorcheh, Soheila Rahgozar, Daryush Talei

**Affiliations:** ^1^ University of Isfahan Isfahan Iran; ^2^ Medicinal Plants Research Center Shahed University Tehran Iran

**Keywords:** 6‐shogaol, acute lymphoblastic leukaemia, combination therapy, drug resistance, ginger extract

## Abstract

Combination therapies, using medicinal herbs, are broadly recommended to attenuate the chemotherapy adverse effects. Based on our previous findings considering the anti‐leukaemic effects of ginger extract on acute lymphoblastic leukaemia (ALL) cells, the present study was aimed to investigate the anti‐cancer role of this pharmaceutical plant on ALL mice models. Moreover, we worked towards identifying the most anti‐leukaemic derivative of ginger and the mechanism through which it may exert its cytotoxic impact. In vivo experiments were performed using five groups of six C57BL/6 nude mice, and the anti‐leukaemic activity of ginger extract alone or in combination with methotrexate (MTX) was examined. Results showed increased survival rate and reduced damages in mice brain and liver tissues. Subsequently, MTT assay demonstrated synergistic growth inhibitory effect of 6‐shogaol (6Sh) and MTX on ALL cell lines and patients primary cells. Eventually, the molecular anti‐neoplastic mechanism of 6Sh was evaluated using Bioinformatics. Flow cytometry illustrated 6Sh‐mediated apoptosis in Nalm‐6 cells confirmed by Western blotting and RT‐PCR assays. Further analyses exhibited the generation of reactive oxygen species (ROS) through 6Sh. The current study revealed the in vivo novel anti‐leukaemic role of ginger extract, promoted by MTX. Moreover, 6‐shogaol was introduced as the major player of ginger cytotoxicity through inducing p53 activity and ROS generation.

## 1 INTRODUCTION

Leukaemia is the most common cancer among children and is accounted for 28% of all cases.^1^ Acute lymphoblastic leukaemia (ALL) is the most frequently diagnosed type of leukaemia among children.^2^ This heterogeneous disease originates from T‐ or B‐cell lineage, in which approximately 85% are B‐ ALL.^3^ Despite advances in ALL treatment and increasing 5‐year survival from 41% in the mid‐1970s to 70% for the period of 2005 to 2011,^4^ 15%‐20% of patients with this malignancy, will suffer from relapse.^5^

Methotrexate (MTX), a well‐known chemotherapeutic agent,^6^ inhibits dihydrofolate reductase (DHFR). DHFR is essential for the biosynthesis of purines; therefore, MTX impairs de novo synthesis of DNA.^7^ Although chemotherapy is a common treatment for cancer, it has a wide range of side effects, including myelosuppression, immunosuppression, mucositis, hepatotoxicity, neurotoxocity, hair loss, nausea and vomiting.^8^ Medicinal plants alone or in combination with chemotherapeutic drugs may reduce chemotherapeutic agent’s adverse side effects.^9^, ^10^

Ginger, the rhizome of the Zingiber officinale, has been widely used for its pharmacological properties including anti‐tumour, anti‐microbial, anti‐inflammatory, anti‐diabetic, anti‐obesity, hepatoprotective and gastroprotective effects.^11^ Gingerols and shogaols are major polyphenols in fresh and dried ginger, respectively. These compounds are responsible for different bioactivities of ginger.^12^ Investigating the cytotoxic effect of gingerols and shogaols on human A549, SK‐OV‐3, SK‐MEL‐2, HCT15 and PC3 tumour cells has shown that 6‐shogaol (6Sh) possesses the most potent inhibitory effect among these derivatives.^13^, ^14^ In addition, different studies indicated that growth inhibitory effect of 10‐gingerol is more than other gingerols in cancer cells, including melanoma, promyelocytic leukaemia, breast, lung, ovarian and colon cancers.^13^, ^15^, ^16^, ^17^, ^18^ However, none of these derivatives are proved to have impact on ALL patients. Several pathways are suggested through which ginger derivatives may apply their anti‐cancer effects. There are published data indicating the role of ginger derivatives in increasing the expression levels of the tumour suppressor protein p53.^19^, ^20^ Moreover, 6Sh is claimed to target cell‐cycle arrest by decreasing cyclinD1/3, survivin and cMyc in prostate and non‐small cell lung cancers.^21^, ^22^ Interestingly, it has been shown that 10‐gingerol may induce apoptosis and inhibit proliferation through PI3K/AKT, AMPK, mTOR and p38MAPK in cervical and breast cancer cells, respectively.^20^, ^23^ Fatty acid synthase (FASN) is another key regulator which may be responsible for leukaemia drug resistance. According to our previous study *FASN* expression levels are upregulated in ALL drug resistant children, and they may be reduced by cells treating with ginger extract.^24^ It would be interesting to know which ginger derivative could play role in the *FASN* molecular pathway. Moreover, it is shown that *FASN* overexpression may inhibit TNF‐α expression, caspase 8 and NF‐κB activation in several cancer cells.^25^ However, the exact plant derivative which may influence its apoptotic effect is yet to be identified.

The anti‐cancer effect of ginger extract was documented in ALL cell lines and primary cells by our group.^26^ However, its inhibitory effect on ALL animal models was remained unknown. In the current study, we examined the anti‐leukaemic activity of ginger extract, as a single agent or combined with MTX, on the cell line‐derived xenograft mouse models. Additionally, the cell growth inhibitory effects of 6‐shogaol and 10‐gingerol were compared. Subsequently, the most anti‐leukaemic ginger derivative was selected, and combined therapies with MTX were investigated on the Nalm‐6 cell line and ALL primary cells. Finally, bioinformatics and in vitro assays were performed to identify the molecular mechanisms through which, 6‐shogaol may exert its cytotoxic impact on leukaemic cells. The rationale for selecting MTX in this project was its broad clinical use in every stages of ALL treatment and to validate our interesting in‐vitro results of anti‐MTX resistant effects of ginger extract ^26^ in ALL mice models.

## 2 MATERIALS AND METHODS

### 2.1 Reagents

Ginger extract was obtained from Shaanxi Zhengsheng Kangyuan Bio‐medical Co., Ltd. 6‐Shogaol was purchased from Adooq Bioscience. 10‐gingerol, phosphate‐buffered saline (PBS), mouse anti‐p21 monoclonal antibody, 2′,7′‐Dichloro‐fluorescin diacetate (DCFH‐DA) and N‐acetyl‐l‐cysteine (NAC) were bought from Sigma‐Aldrich. MTX, mouse anti‐p53 monoclonal antibody and mouse anti‐β actin monoclonal antibody were acquired from Santa Cruz Biotechnology, Inc. Dimethyl‐sulfoxide (DMSO) was from Cinnagen. Roswell Park Memorial Institute‐1640 (RPMI1640), foetal bovine serum (FBS) and penicillin streptomycin (Pen Strep) were obtained from Bioidea. 3‐(4,5‐dimethylthiazol‐2‐yl)‐2,5‐diphenyltetrazolium bromide (MTT) was from Atocel. l‐glutamine was bought from Gibco and FITC Annexin‐V Apoptosis Detection Kit with PI was purchased from BioLegend. Ficoll–Hypaque was obtained from Inno‐train and TRIzol reagent was from Invitrogen. PrimeScript™ RT reagent Kit was from Takara. Protein ladder and goat anti‐mouse immunoglobulins/HRP were from Thermo Fisher Scientific and Dako Denmark A/S respectively. Nitrocellulose membrane and Amersham ECL Prime Western Blotting Detection Reagent were purchased from GE Healthcare.

### 2.2 Animal model and treatments

Four‐ to six‐week‐old female athymic nude mice (C57BL/6 nude) were purchased from Pasteur Institute of Iran. Animals were housed in the Specific‐Pathogen‐Free Animal Laboratory, Department of Biology, University of Isfahan. The protocol was approved by the university’s Ethics Committee on Animals Handling (Permission number: IR.UI.REC.1396.056). Mice were kept in the laboratory for two weeks without testing for acclimation to the new environment. Transplantation was initialized by giving mice 300 mg/kg cyclophosphamide intraperitoneally. Three days later, they were injected with 15 × 10^6^ CCRF‐CEM cells in 100 μL FBS, subcutaneously. In order to confirm leukaemia engraftment, flow cytometry was performed on mice blood samples by using antibodies against CCRF‐CEM cell‐line CD markers. Separate treatment protocols were started the day after engraftment. Mice were divided into four groups of six. Two groups were injected intraperitoneally with 80 mg/kg ginger extract five times a week or 5 mg/kg MTX once a week. The third group was given both of the aforementioned regimens and the fourth group was treated with vehicle. Mice were sacrificed two months post‐transplantation. Liver, brain and bone marrow were collected following slaughter. Liver and brain tissues were stained with H&E, and bone marrow samples were stained with Wright–Giemsa stains along with the conventional methods.

### 2.3 Cell lines

CCRF‐CEM (T‐ALL) and Nalm‐6 (B‐ALL) human cell lines were obtained from Pasteur Institute. R‐CCRF‐CEM (a T‐ALL subline resistant to MTX) (National patent number: 98824) and RN95 (a B‐ALL cell line derived from an Iranian female child with relapsed ALL) (National patent number: 100281) cell lines were developed in‐house. Cells were maintained in RPMI1640 containing 10% heat‐inactivated FBS and 1% penicillin/streptomycin. For Nalm‐6 and R‐CCRF‐CEM cell lines, culture medium was additionally supplemented with 1% l‐glutamine and 1.2 μmol/L MTX, respectively. Cells were incubated at 37°C and an atmosphere of 5% CO_2_.

### 2.4 Patient and control sampling

10 children with ALL who referred to Sayed‐ol‐Shohada Hospital from 2019 to 2020 were included in the present study. The project was permitted by the Ethics Committee of University of Isfahan under the agreement number IR.UI.REC.1398.009 and conducted according to the Declaration of Helsinki guidelines. Two to five millilitres of heparinized bone marrow sample or peripheral blood were collected from ALL patients and sent on ice to the cellular and molecular biology laboratory of University of Isfahan. Patient primary cells and control mononuclear cells were isolated by Ficoll–Hypaque density gradient centrifugation method, according to the manufacturer’s protocol and cultured in RPMI1640 containing 20% FBS and 1% l‐glutamine.

### 2.5 Cell treatment

Cell lines were cultured in 96‐well tissue culture plates at a density of 2 × 10^4^ per well, in 100 μL FBS supplemented media culture. To validate the anti‐leukaemic effect of ginger derivatives, cells were treated with 50 μL increasing concentrations of 6‐shogaol (10‐200 μmol/L) and 10‐gingerol (10‐200 μmol/L) in 0.4% DMSO. For the combination treatments, cells were incubated with 25 μL of the selected ginger derivative and 25 μL MTX. 10 × 10^4^ patient primary cells were cultured in 96‐well plates and treated with 200 μmol/L 6Sh or 0.1 μmol/L MTX, alone or in combination, according to our previous published study.^26^

### 2.6 In vitro viability/proliferation assays

To determine cell proliferation and viability, MTT assay was used. At the end of the incubation time, 10 μL of MTT solution (5 mg/mL) was added to wells. Following incubation at 37°C for 3hours, supernatant was removed and formazan crystals were solubilized by adding 100 μL DMSO into each well. Absorbance was recorded at 492 nm by using a stat fax‐2100 microplate reader (Awareness Technology, Inc).

### 2.7 Functional enrichment and pathway analysis

Sufficient literature mining was performed to identify all the genes whose expressions were significantly affected by 6Sh. Subsequently, gene‐enrichment and functional annotation analyses were performed using Functional Annotation Tool (the visualization and integrated discovery (DAVID) Bioinformatics database (https://david.ncifcrf.gov/) to gather pathways related to the target genes and proteins.

### 2.8 Apoptosis assay

The FITC Annexin‐V Apoptosis Detection Kit with PI was used to detect cell apoptosis rate. Briefly, 25 × 10^4^ cells/well were suspended in 100 μL Annexin V binding buffer and transfer into a flow cytometry tube. Cells were, then, stained with 1.5 μL FITC Annexin‐V and 2 μL PI solutions, and incubated at room temperature (25°C) for 15 minute in the dark. Subsequently, 400 μL ice‐cold Annexin V binding buffer was added to each tube and Annexin V and/or PI‐positive cells were counted using a BD FACSCalibur Flow Cytometer. Data were analysed by Cell Quest Pro (BD Biosciences) and FlowJo software version 7.6.1 (Tree Star Inc., Ashland, OR).

### 2.9 Western blot analysis

Western blot analysis was performed to investigate the potential role of p53 and p21 proteins in the cytotoxic effects of 6Sh. Lysis buffer was used to extract total proteins from cells, and Bradford protein assay was done to determine the concentration of proteins. 30 μg of protein extracts were separated on a gradient polyacrylamide gel and transferred from the gel to a nitrocellulose membrane. Membrane was blocked with 5% skimmed milk in 1 × TBS/Tween solution for 1 hour followed by incubation with primary antibodies for 1.5 hour at room temperature and overnight at 4°C. Mouse anti‐p53, anti‐p21 and anti‐β actin monoclonal primary antibodies were used at 1:250, 1:100 and 1:250 dilutions, respectively. After washing with 1 × TBS/Tween solution, membrane was incubated with secondary antibody goat anti‐mouse immunoglobulins/HRP (1:1000) at room temperature for 1 hour. Finally protein detection was performed using sensitive radiology films in a dark room using ECL Prime Western Blotting Detection Reagent.

### 2.10 RNA isolation and real‐time PCR

Extraction of total RNA, DNase treatment and cDNA synthesis were performed using TRIzol reagent and PrimeScript™ RT reagent Kit (Takara) in accordance with the manufacturer’s protocols, respectively. Gene expression was determined using Ampliqon RealQ Plus Master Mix Green high ROX™. All PCR reactions were done in triplicates during two independent experiments by a Chromo4™ system (Bio‐Rad). The sequences of the primers utilized in the current study are mentioned in Table 1. Relative quantifications of gene expressions were calculated by 2^−ΔΔ^ *^C^* ^t^ method.

**TABLE 1:** Primer sequences of *PUMA*, *FASN* and *GAPDH*

Gene	Primer sequence (5' to 3')	Primer length (base pairs)	Amplicon length (base pairs)	*T*_m_	GC%
PUMA	F: GACCTCAACGCACAGTACGAG	21	98	60.99	57.14
	R: AGGAGTCCCATGATGAGATTGT	22		58.61	45.45
FASN	F: CCGCTTCCGAGATTCCATCCTACGC	25	137	67.13	60
	R: GGATGGCAGTCAGGCTCACAAACG	24		66.19	58.3
GAPDH	F: GCCCCAGCAAGAGCACAAGAGGAAGA	26	106	68.64	57.69
	R: CATGGCAACTGTGAGGAGGGGAGATT	26		66.38	53.85

Abbreviations: F, forward; FASN, fatty Acid Synthase; GAPDH, glyceraldehyde 3‐phosphate dehydrogenase; PUMA, p53 upregulated modulator of apoptosis; R, reverse; *T*m, melting temperature.

### 2.11 Detection of the ROS

In order to detect ROS production, cells (5 × 10^5^ cell/well) were incubated with 500 μL FBS‐free medium containing 40 μmol/L DCFH‐DA in the dark. 15 minute later, 500 μL ice‐cold PBS were added to each well and the fluorescence was monitored by a Partec CyFlow ML Flow Cytometer supported by FloMax software in the FL‐1 channel. Data were plotted on histograms using FlowJo software version 10.

### 2.12 Combination index

Drug interaction was measured using the combination index (CI), where CI˂0.9, 0.9 ˂CI<1.1, and CI>1.1 show synergistic, additive and antagonistic effects, respectively. CI value was calculated according to the equation stated below:

$$CI=\frac{{\left(D_{x}\right)}_{com1}}{{\left(D_{x}\right)}_{1}}+\frac{{\left(D_{x}\right)}_{com2}}{{\left(D_{x}\right)}_{2}},$$

(*D*_x_)_com1_ (or (*D*_x_)_com2_) was the drug concentration in the combination treatment that inhibited×% proliferation, and (*D*_x_)_1_ (or (*D*_x_)_2_) was the drug concentration 1 (or 2) alone, that triggered the same percentage inhibition.

### 2.13 Statistical analysis

Image J software was used for histomorphometric analyses. In this software, haematoxylin and eosin pathological images were converted to RBG format and the percentage of tissue damages were calculated by determining a specific threshold. All statistical analyses including one‐way ANOVA and *t*‐tests were performed using the GraphPad Prism version 6 software (GraphPad Prism Inc). All data were reported as mean ± standard error of mean (SEM). *P* ˂ .05 was considered to be statistically significant.

## 3 RESULTS

### 3.1 Effects of ginger extract, alone or in combination with methotrexate on ALL mouse models

**FIGURE 1:**
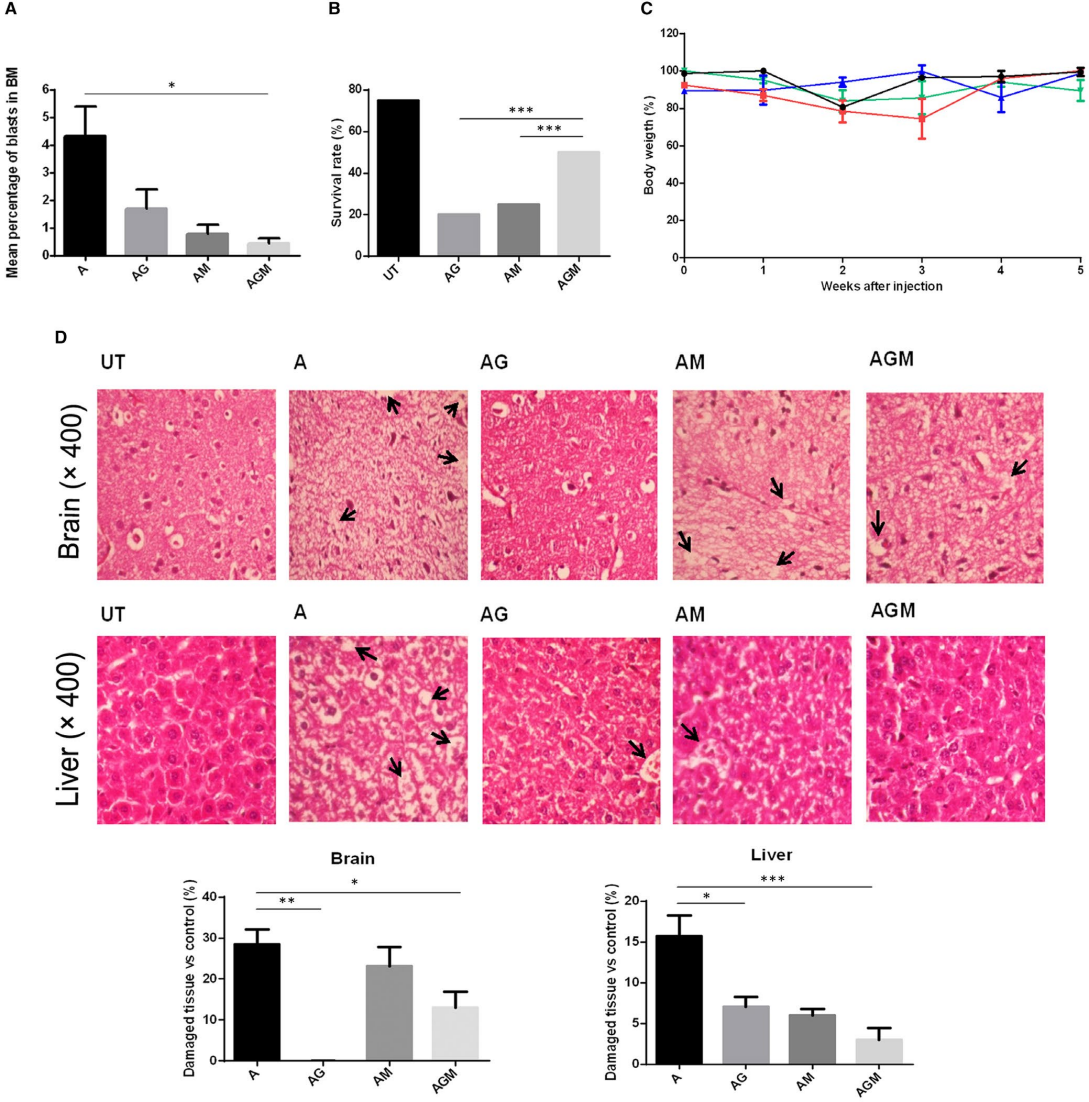
The outcome of ginger extract treatment, alone or in combination with MTX, in the mouse models of ALL. After 14 d of adaptation, C57BL/6 nude mice were intraperitoneally injected with cyclophosphamide (300 mg/kg). 72 h later, CCRF‐CEM cell transplantation was performed subcutaneously and leukaemia engraftment was authenticated by flow cytometry (Data not shown). One group of six remained untransplanted (UT). ALL mice were divided into four groups of six. Treatment was administered by intraperitoneal injections of 80 mg/kg ginger extract (AG), 5 mg/kg MTX (AM) and a combination of 80 mg/kg ginger extract and 5 mg/kg MTX (AGM). The fourth group was treated with vehicle (A). 60 d after transplantation, mice were sacrificed. (A,B,C) Mice treated with combined concentrations of ginger extract and MTX showed significant decrease in the number of bone marrow (BM) blasts, and their survival rate was increased compared with those which got single treatments. There was no significant difference in percentage body weight between different groups of transplanted mice. (D) Brain and liver tissue sections of drug‐ and vehicle‐treated ALL mice models were fixed in 10% formalin, paraffin‐embedded and stained with haematoxylin and eosin dyes. Results showed that single and combined treatment of the ALL mice models with ginger extract showed decreased histopathological damages in the brain and liver tissues. Some areas of damage are marked by arrows. For statistical analysis, Image J software was used. Results were consistent with pathological interpretation of the tissues. Three sections were examined in each mouse. Values are mean ± SEM, *P < .05, **P < .01, ***P < .001

After a two‐week adaptation period, mice were injected with cyclophosphamide and then with CCRF‐CEM cells. Transplantation was confirmed using flow cytometry (Data not shown). Subsequently, mice were treated with ginger extract and MTX, alone or in combination. Two months after transplantation, mice were sacrificed. The malignant cells percentage invasion in bone marrow was calculated using Wright‐Giemsa Stain protocols. Results showed significant decrease in bone marrow blasts when mice were treated with ginger extract/MTX compared with the vehicle‐treated mice [0.45% ± 0.17% vs 4.33% ± 1.07% (mean ± SEM), respectively, *P* < .05] (Figure 1A). Moreover, combined treatment with ginger extract and MTX prolonged significantly the ALL mice percentage survival rate compared with the single treatments (50% vs 20% and 25%, respectively, *P* < .001) (Figure 1B). Loss of body weight was not evident in any of the animal groups (Figure 1C).

Haematoxylin/Eosin staining demonstrated less damage in brain and liver tissues of mice treated with ginger extract/MTX compared with the vehicle‐treated group [13.02% ± 3.78% vs 28.53% ± 3.61% (mean ± SEM), *P* < .05, and 2.97% ± 1.49% vs 15.72% ± 2.49% (mean ± SEM), *P* < .001, respectively]. Interestingly, single treatment with ginger extract showed only 0 ± 0.06% brain damage (Figure 1D).

### 3.2 Effects of ginger derivatives on ALL cell lines viability

**FIGURE 2:**
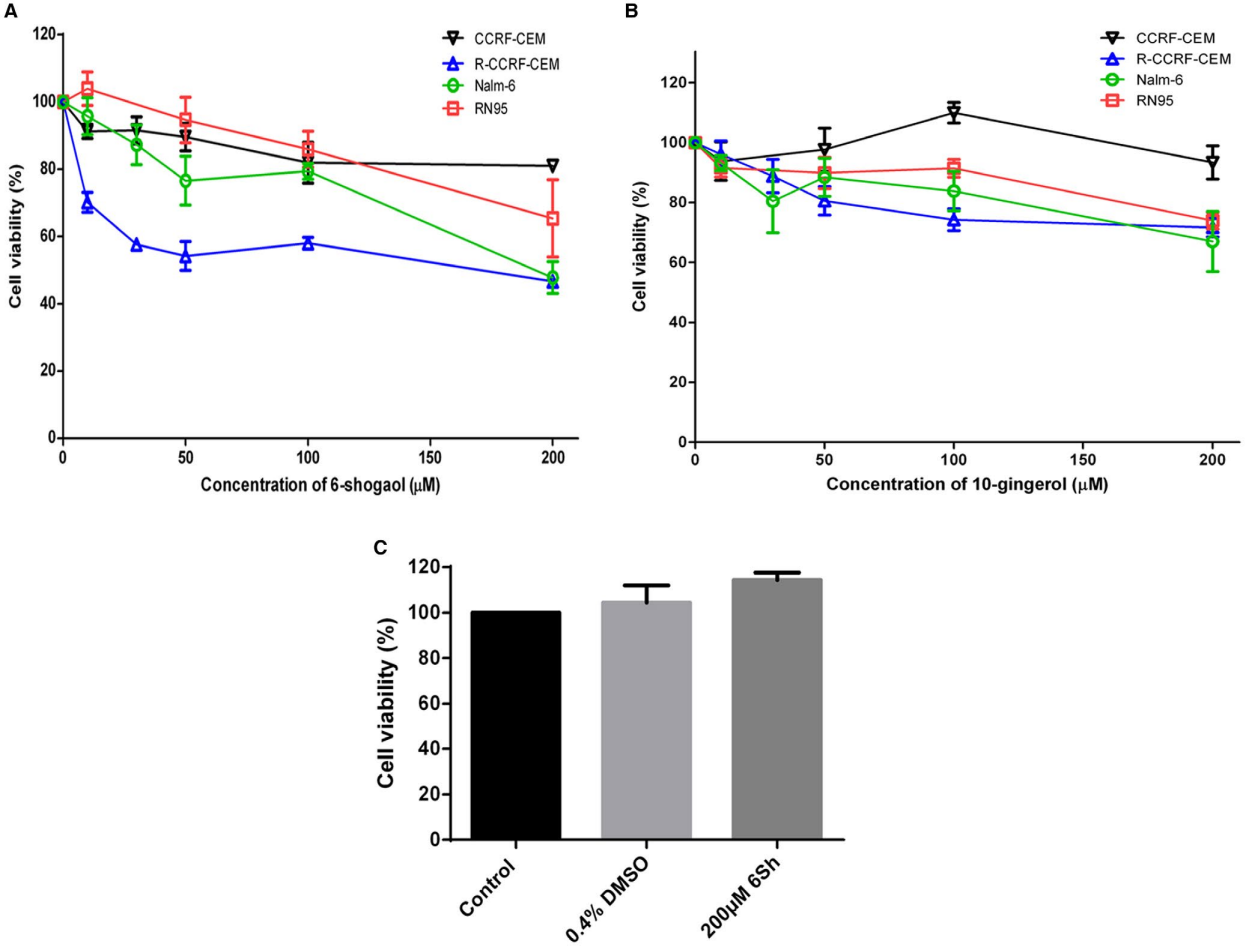
FIGURE(A,B) The growth inhibitory effects of 6‐shogaol and 10‐gingerol on four different leukemic cell lines. Human leukaemic cell lines were treated with increasing concentrations of 6Sh and 10‐gingerol for different time points related to their growth curves. The percentage of cell viability was decreased in a dose‐dependent manner, especially for Nalm‐6 and R‐CCRF‐CEM cell lines. Among these two cell lines, Nalm‐6 was chosen for subsequent experiments since the related immunophenotype was more prevalent among children with ALL. Furthermore 200 µmol/L 6Sh was selected due to its proximity to the half‐maximal concentrations of the proliferation inhibition (IC50) of 6Sh on Nalm‐6 cell line. (C) Neither 6‐shogaol (6Sh), nor its solvent affect the survival of normal mononuclear cells (MNCs). Mononuclear cells were isolated from three volunteers. 10 × 10^4^ cells were seeded per well. Following 48 h treatment with 0.4% DMSO or 200 µmol/L 6Sh, the viability of MNCs was determined using MTT assay. For the control group, cells were incubated with RPMI1640 alone. Data were reported as mean ± SEM of three separate experiments. Each experiment was performed in triplicates. Black = CCRF‐CEM cell line, blue = R‐CCRF‐CEM cell line, green = Nalm‐6 cell line and red = RN95 cell line

Two T‐ALL cell lines; one purchased, CCRF‐CEM; and one in‐house generated MTX‐resistant subline, R‐CCRF‐CEM, and two B‐ALL cell lines; one purchased, Nalm‐6; and one patient‐derived in‐house generated cell line, RN95, were chosen to evaluate the impact of the ginger derivatives: 6‐shogaol and 10‐gingerol on ALL cell viability. Plates were coated with 2 × 10^4^ cells per well in triplicates. Cell viability was assessed using MTT assays after 72 hours (for CCRF‐CEM, R‐CCRF‐CEM and RN95) and 96 hours (for Nalm‐6). Incubation times were chosen according to the cell lines doubling times (Figure 2A,B). The half‐maximal concentration of proliferation inhibition (IC50) for each of the abovementioned derivatives is presented in Table 2. Since Nalm‐6 was the most vulnerable cell line to ginger derivatives at high concentrations (Figure 2A,B), and B‐ALL is the most frequent immunophenotype among paediatric leukaemias ^3^, Nalm‐6 was chosen as the ALL‐cell line for further experiments. On the other hand, among the two examined ginger derivatives, 6‐shogaol showed more cytotoxicity on ALL cell lines therefore selected for additional investigations (Table 2, Figure 2A). To evaluate the impact specificity of 6‐shogaol on leukaemic cells, the possible negative effect of this component was measured on fresh normal peripheral mononuclear cells (MNCs) using MTT assay. 10 × 10^4^ MNCs per well were seeded into 96‐well plates in triplicate and treated with 200 µmol/L 6Sh or 0.4% DMSO (as its solvent) for 48 hours. Normal MNCs did not show sensitivity to proliferation inhibition induced by neither 200 µmol/L 6Sh nor 0.4% DMSO (Figure 2C).

**TABLE 2:** The anti‐leukemic effect of ginger derivatives on different paediatric ALL cell lines

Compounds	IC50 (µmol/L)			
	CCRF‐CEM	R‐CCRF‐CEM	RN95	Nalm‐6
6‐shogaol	>200	170 ± 5	>200	191.33 ± 2.96
10‐gingerol	>200	>200	>200	>200

Note

Data are expressed as mean value ± SEM, IC50 = 50% inhibition concentration, CCRF‐CEM = T‐ALL cell line, R‐CCRF‐CEM = In‐house T‐ALL subline resistant to methotrexate, Nalm‐6 = B‐ALL cell line, RN95 = in‐house B‐ALL cell line derived from an Iranian female child with relapsed acute lymphoblastic leukaemia.

### 3.3 Effect of the combination of 6‐shogaol with methotrexate on the B‐ALL cell line

**FIGURE 3:**
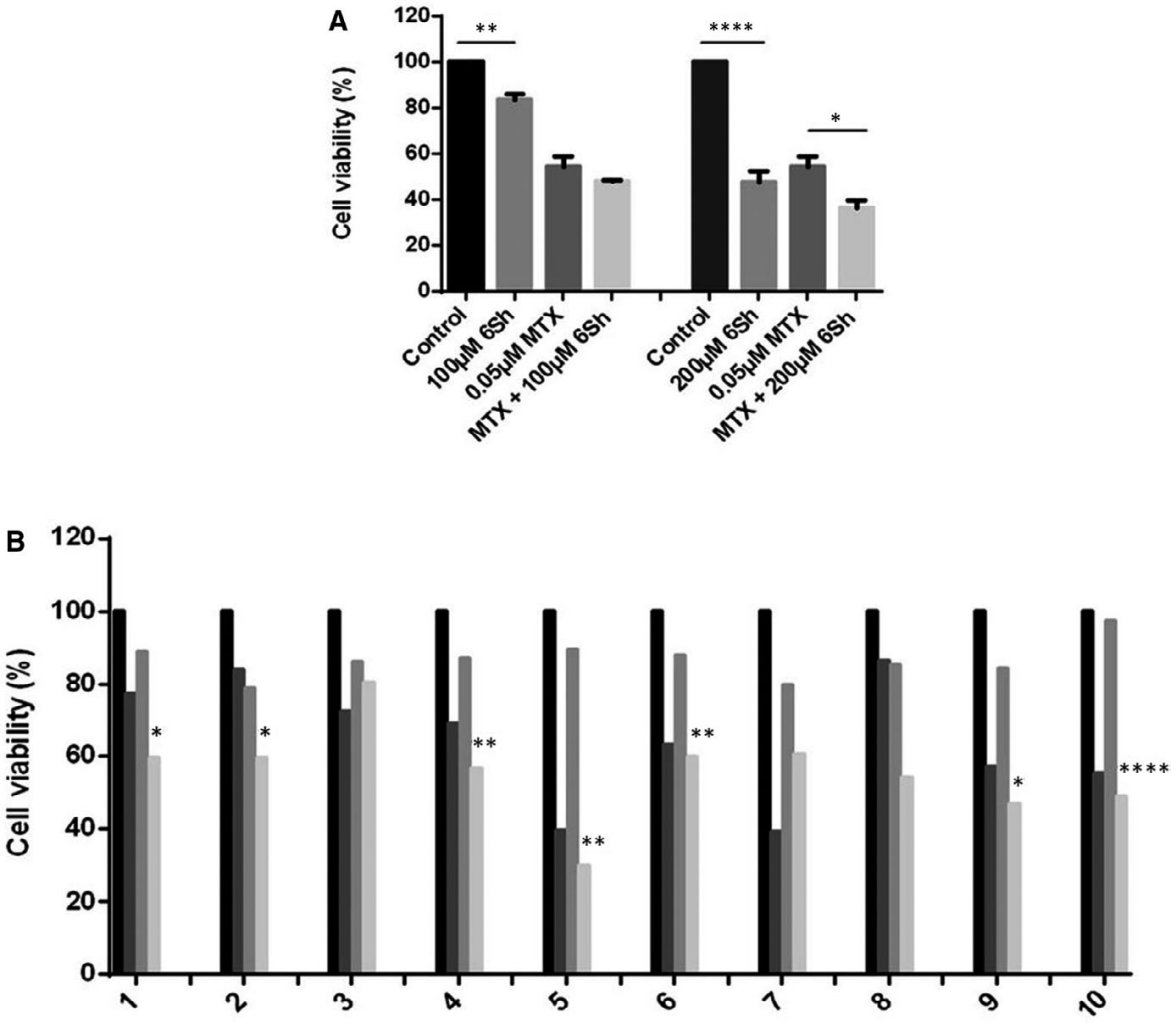
Both single and combined concentrations of 6‐shogaol (6Sh) and methotrexate (MTX) show growth inhibitory effects on Nalm‐6 cell line and patients’ primary cells. (A) Cells were seeded in 96‐well flat bottom plates and incubated with 0.05 μmol/L MTX alone and in combination with 100 µmol/L and 200 µmol/L 6Sh for 96 h. Cell viability was assessed using MTT assay. Results showed that the cytotoxic effect of MTX alone was significantly less than that of its combination with 200 µmol/L 6Sh. B, Combined treatment of ALL patients’ primary cells with 6‐shogaol (6Sh) and methotrexate decreases cells viability. Mononuclear cells were isolated from patients’ peripheral blood or bone marrow, at diagnosis. Cells were then; seeded into 96 well plates followed by incubation with single or combined concentrations of 200 µmol/L 6‐shogaol and 0.1 μmol/L MTX for 48 h. Cells viability was measured using MTT assays. Numbers are representatives of diverse patients. Columns for each patient demonstrate vehicle‐treated cells, cells treated with 200 µmol/L 6Sh, 0.1 μmol/L MTX and combined 200 µmol/L 6Sh/0.1 μmol/L MTX, respectively. Comparisons between the third and fourth groups were shown by asterisks. Each experiment was performed in quadruplicates. Values are mean ± SEM, *P < .05, **P < .01, ****P < .0001

To investigate the cytotoxic effect of 6Sh in combination with MTX on Nalm‐6 cell line, the IC50 of MTX alone was primarily calculated using MTT assay as 0.05 ± 0.01 µmol/L (mean ± SEM) (Data not shown). Subsequently, cells were treated with single or combined concentrations of 0.05 µmol/L MTX and 100 µmol/L or 200 µmol/L 6Sh for 96 hours. Results demonstrated that 6Sh/MTX had significantly higher growth inhibition effect compared with MTX alone [36.54% ± 3.03% vs 51.93% ± 7.76%, respectively) (mean ± SEM, *P* < .05)] (Figure 3A).

The calculated combination index (CI) from dose‐effect data of single and combination treatments, indicated synergistic effect for 6Sh and MTX on Nalm‐6 (CI = 0.76).

### 3.4 Effect of 6Sh/MTX combination treatment on patient primary cells

The anti‐proliferative effect of 6Sh/MTX combination was studied on the fresh primary malignant samples collected from eight de novo and two relapsed patients with childhood ALL (Table 3). Isolation of mononuclear cells from patients’ whole blood/bone marrow, at diagnosis, was accomplished by using density gradient Ficoll media. 10 × 10^4^ cells were seeded into the 96‐well plates and treated with 200 µmol/L 6Sh, 0.1 µmol/L MTX and the combination of 6Sh/MTX for 48 hours. Cell viability was then calculated using MTT assays. Inhibition of the cell proliferation was observed in 7 out of 10 6Sh/MTX‐treated sample patients compared with samples treated with MTX alone [51.55% ± 4.15% vs 87.56% ± 2.13% (mean ± SEM, n = 1), *P* < .05] (Figure 3B).

**TABLE 3:** Clinical and molecular characteristics of the children with ALL, participated in the study

Case No	Patient’s condition	Source (WB/ BM)	Sex	Age(y)	ALL subtype	ALL related translocations including: T (4;11)/KMT2A‐AFF1 T (9;22)/BCR‐ABL1 T (1;19)/TCF3‐PBX1 T (12;21)/ETV6‐RUNX1
1	Relapsed	PB	M	2.5	Pre‐B	Negative
2	Relapsed	BM	M	6	Pre‐B	Negative
3	New case	PB	F	0.5	Pre‐B	Negative
4	New case	PB	F	4	Pre‐B	Negative
5	New case	PB	M	5	Pre‐B	Negative
6	New case	PB	F	6	Pre‐B	Negative
7	New case	PB	M	13	Pre‐T	Negative
8	New case	BM	F	4	Pre‐B	Negative
9	New case	PB	F	7	Pre‐T	Negative
10	New case	PB	M	5	Pre‐B	Negative

Abbreviations: BM, bone marrow; F, female; M, male; PB, peripheral blood.

### 3.5 Pathway analysis through DAVID database

**FIGURE 4:**
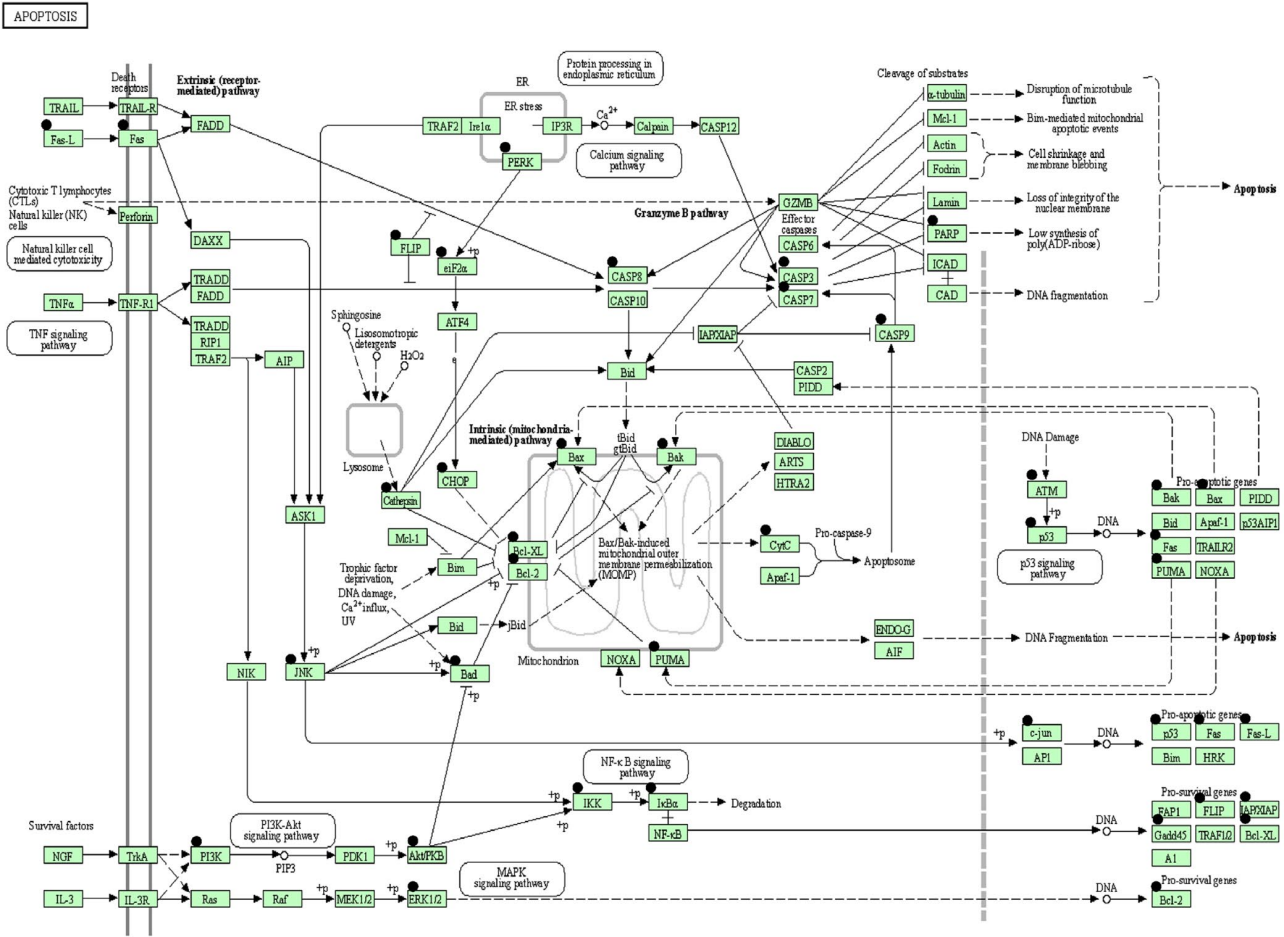
FIGURE6‐Shogaol induces apoptosis through several pathways. Apoptosis pathways were provided by the Database for Annotation, Visualization and Integrated Discovery (DAVID) tool (https://david.ncifcrf.gov/). The black circles above each gene represent the genes whose expression are affected by 6‐shogaol. White box = map, White circle = chemical, compound, DNA or other molecules, +P = phosphorylation, 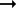 = activation/expression, 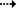 = indirect link or effect, 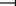 = inhibition/repression

The 6‐shogaol regulated target genes and proteins were identified by studying literature reviews and analysed using DAVID Bioinformatics database to find 6Sh related pathways. Results demonstrated that 6Sh induces apoptosis through different pathways including intrinsic and extrinsic apoptosis pathways, calcium signalling pathway, protein processing in endoplasmic reticulum and p53 signalling pathways (Table 4, Figure 4).

**TABLE 4:** The apoptosis related genes and proteins regulated by 6‐shogaol

Genes/Proteins^a^	Types of cancer	References
Fas, Fas‐L, CASP3, CASP9, PARP, CytC, Bcl‐2, Bcl‐XL	Colorectal carcinoma	^40^
FLIP	Renal carcinoma	^41^
eiF2α, Cathepsin	Histiocytic lymphoma	^42^
CASP3, CASP8, CASP9, PARP, Bcl‐2, Bcl‐XL, JNK, ERK1/2, IAP	Breast cancer	^43^
CASP3, CASP7, PARP	Non‐small cell lung cancer	^22^
Bax, Bcl‐2, CytC, CASP3,CASP9	Laryngeal cancer	^44^
p53, PUMA, Bak, Bax, CASP3, CASP9, CytC	Lung Cancer Cell	^45^
ERK1/2, JNK	Hepatoma	^46^
Bcl‐2, CASP3, PARP, IAP, XIAP	Pancreatic Cancer	^47^
*p53*, *Gadd45*, *Bcl‐2*, *CASP3*, *CASP8*, *CASP9*, *Fas‐L*, *ATM*, *Bad*	Colon Cancer	^19^
ERK1/2, JNK, PI3K, Akt, IκBα	Hepatocarcinoma	^48^
*Bax*, *Bcl‐2*, IκBα	Prostate Cancer	^21^
JNK, IκBα, IKK, c‐jun	Breast cancer	^49^
Bcl‐2, JNK	Colon cancer	^50^
CHOP, eiF2α, PERK	Hepatocellular Carcinoma	^51^

^a^ Italics show genes.

### 3.6 Effect of 6‐shogaol on inducing apoptosis

**FIGURE 5:**
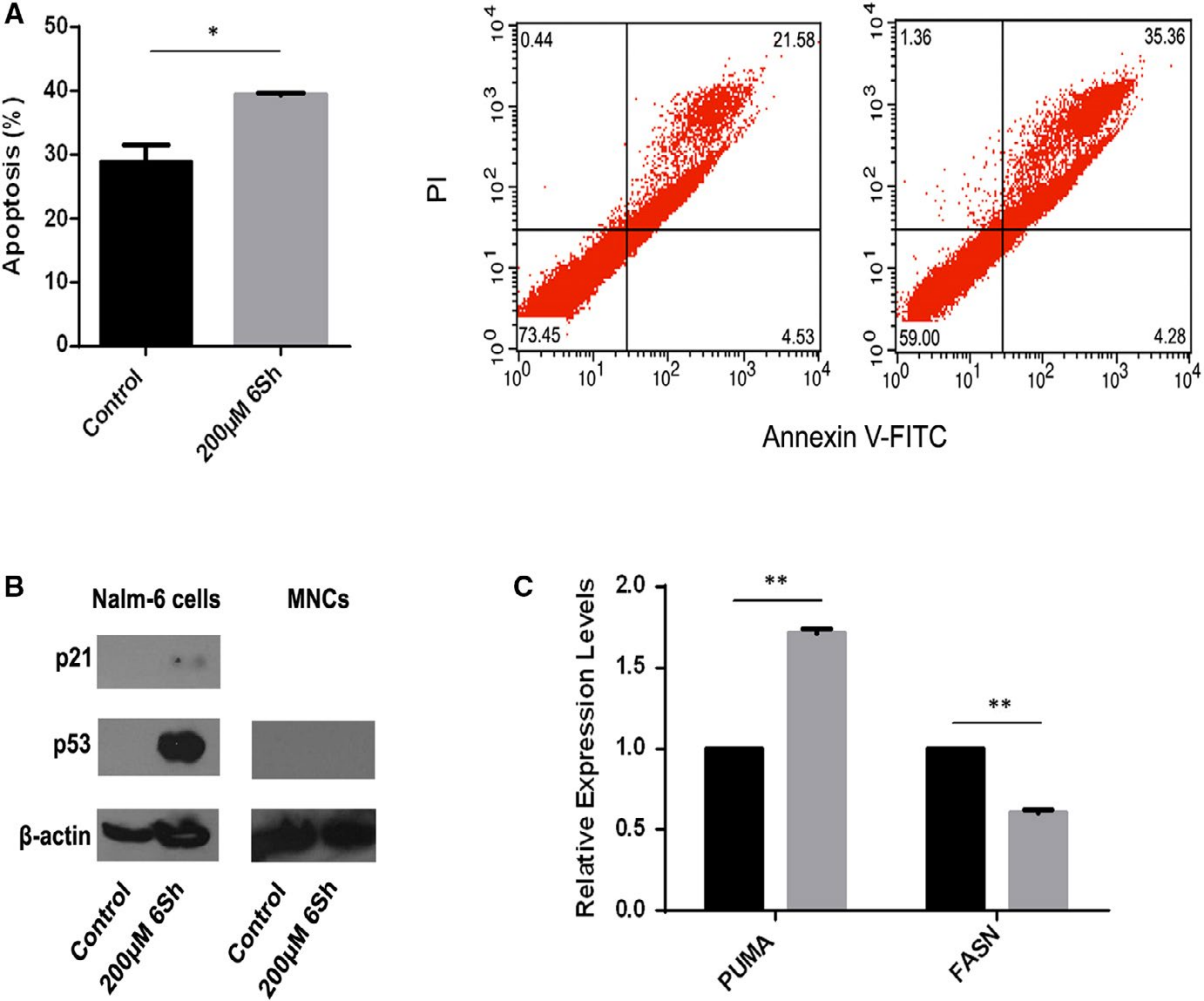
Six‐shogaol (6Sh) induces apoptosis on B‐ALL leukemic cells. A, Nalm‐6 cells were seeded into 96 microtiter plates and incubated with 200 µmol/L 6Sh for 96 h. Apoptosis was calculated using Annexin‐V/PI apoptosis detection kit and flow cytometry. Percentage apoptosis induced by 6Sh was significantly higher than that of the vehicle‐treated cells. Data are represented as means ± SEM of three separate experiments. Two representatives of flow cytometry analyses are demonstrated for the control cells (left) and those treated with 200 μmol/L 6Sh (right). B, Six‐shogaol persuades cell‐cycle arrest through activation of p53 and p21. Nalm‐6 cell and MNCs lyses and protein extractions were performed after treatment with or without 200 µmol/L 6Sh for 48 h. Western blot analysis of p53 and p21 protein expressions demonstrated that 6Sh increases p53 and p21 in protein levels in Nalm‐6 cells but not MNCs. C, Six‐shogaol induces apoptosis by modulating the expression levels of *PUMA* and *FASN* genes. Nalm‐6 cell line was treated with 200 µmol/L 6Sh for 96 h (or 12 h for *FASN*). Subsequently, total RNA was extracted using TRIzol reagent. The mRNA expression levels of *PUMA* and *FASN* genes were then assessed using real‐time PCR. Values are mean ± SEM of two independent experiments. Each experiment was performed in triplicates,*P < .05, **P < .01

To evaluate the given information by bioinformatics data analysis and investigate whether 6Sh could trigger apoptosis in Nalm‐6, cells were treated with or without 200 μM 6Sh for 96 hour. Then, apoptosis was analysed using Annexin‐V/PI apoptosis detection kit and flow cytometry analysis. Results demonstrated that 6Sh induced 10.56% apoptosis in the Nalm‐6 cells [39.43% ± 0.19% apoptosis was observed in 6Sh‐treated cells vs 28.87% ± 2.67% apoptosis demonstrated in the control cells (mean ± SEM, n = 3), *P* < .05] (Figure 5A). Results of the performed trypan blue assay to quantify live cells by labelling dead cells were also consistent with results of the apoptosis assay (Data not shown).

To determine the possible targets through which 6Sh may exert its effects on cell‐cycle arrest and apoptosis, Nalm‐6 cells were seeded into 6‐well plates and treated with 200 µmol/L 6Sh for 48 hours or 96 hours, respectively. Cells total protein was extracted, and Western blot analysis was performed using p53 and p21 primary antibodies. Increased expression levels of p53 and p21 were observed in 6Sh‐treated cells compared with the vehicle‐treated cells. However, Western blot analysis demonstrated that 6Sh did not increase p53 protein expression in MNCs (Figure 5B). On the other hand, total RNA was isolated, cDNA was synthesized and the expression profile of genes involved in apoptosis, including *PUMA* and *FASN,* were investigated using real‐time PCR. The expression level of *PUMA* gene was increased in the treated compared with the vehicle‐treated cells by 1.70 ± 0.03 fold (*P* < .01). In addition, the expression of *FASN* was decreased in Nalm‐6 cells, following treatment with 6Sh for 12 hours, by 0.60 ± 0.01 fold (*P* < .01) (Figure 5C).

### 3.7 Effect of 6‐shogaol on generating reactive oxygen species

**FIGURE 6:**
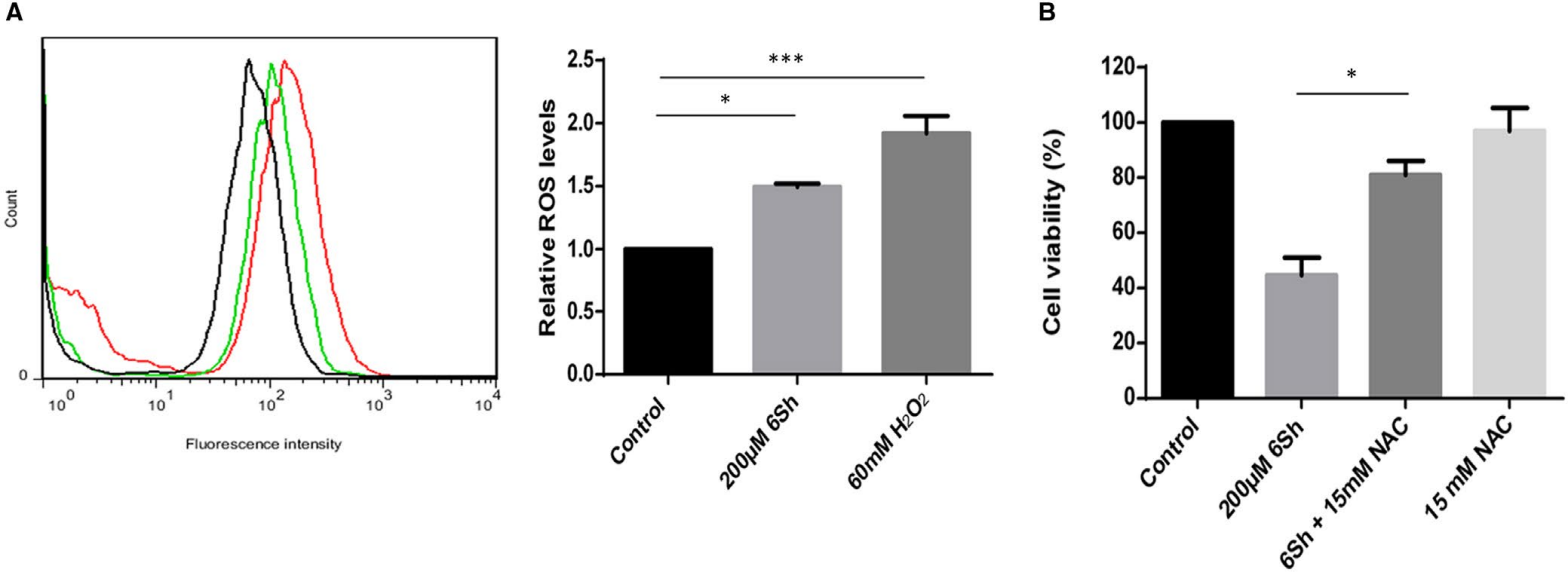
FIGURESix‐shogaol (6Sh) generates reactive oxygen species (ROS) in Nalm‐6 cell line. A, Nalm‐6 cells were treated with 200 µmol/L 6Sh for 9.5 h, then stained with 40 µmol/L 2'7'‐Dichloro‐fluorescin diacetate (DCFH‐DA) for 15 min in dark followed by adding 500‐μL ice‐cold PBS. Cells were then subjected to flow cytometry. 60 mmol/L H_2_O_2_ was used as positive control. Results showed a 50% generation of ROS in cells treated with 6Sh compared with those treated with vehicle. B, The inhibitory effect of N‐acetyl cysteine (NAC), an ROS scavenger, on the cytotoxic impact of 6‐shogaol (6Sh). Nalm‐6 cells were primarily treated with 200 µmol/L 6Sh in the presence or absence of 15 mmol/L NAC for 96 h. Cell viability was assessed using MTT assay. Values are given as the mean ± SEM of two independent experiments performed in triplicates. Black = cells treated with the 6Sh vehicle, green = cells treated with 6Sh, red = cells treated with H_2_O_2_. *P < .05 and ***P < .001

To further explore the mechanism by which 6Sh may exert its impact on cell apoptosis, ROS levels were calculated upon the treatment of Nalm‐6 cells by this pharmaceutical drug. The generated ROS level, as assessed by 2′,7′‐Dichloro‐fluorescin diacetate (DCFH‐DA) staining, was particularly higher in 6Sh‐treated cells than their relative controls [1.49 ± 0.02 vs 1 (mean ± SEM; n = 2), *P* < .05] (Figure 6A). This effect was reversed by the addition of N‐acetyl cysteine as an ROS scavenger (Figure 6B).

## 4 DISCUSSION

The current study revealed increased anti‐cancer effects of MTX when combined with ginger extract in ALL mice models compared with single treatment. This finding was supported by our previously published in vitro experiments.^26^ Moreover, results demonstrated that combination of ginger extract with MTX may reduce the brain and liver damages attributed to chemotherapy (Figure 1). It was currently illustrated that co‐administration of ginger extract and doxorubicin in breast cancer mouse models may increase the mice survival rate and decrease the tumour volume compared with single treatment with doxorubicin.^27^ On the other hand, the healing effects of ginger extract, as a single pharmaceutical drug, were confirmed for non‐cancer pathological conditions including the neuropathological damages produced in diabetics ^28^ and liver lesions induced by cytotoxic drugs.^29^, ^30^

In order to identify the specific derivate of ginger playing the most effective anti‐leukaemic role of this herbal medicine, two of its most active anti‐cancer derivatives were selected according to the review literature ^31^ and examined on different T and B‐ALL cell lines. 6‐Shogaol demonstrated the stronger inhibitory effect on cell proliferation (Figure 2A,B), therefore, chosen for further experiments. Interestingly, 6Sh showed no cytotoxic effect on normal lymphocytes (Figure 2C). Moreover, combination treatments of MTX with 6‐shogaol displayed synergistic cytotoxic effects on Nalm‐6 cell line and patient primary cells (Figure 3). Although MTX is widely known as one of the major chemotherapy drugs administered for paediatric ALL treatment, various undesirable side effects were identified for this agent, including mucositis, nephrotoxicity, hepatotoxicity and encephalopathy.^32^ Discovery of a novel regimen composed of MTX and a herbal component exhibiting stronger anti‐cancer effect would improve response to treatment and decrease the incidence of relapse. Therefore, high‐dose MTX would be less administered and patients would experience less adverse consequences of this chemotherapy drug. This discovery is indisputably important and deserves close inspection.

To investigate the signalling pathway through which 6‐shogaol could exert its cytotoxic impact, bioinformatics analysis using DAVID Functional Annotation Tool was performed. Results determined that 6‐shogaol may contribute to cell death through targeting apoptosis and activating p53 (Figure 4) in the Nalm‐6 cells, where p53 was previously shown to be wild type (unpublished data). Followed by the in vitro quantitative real‐time PCR assays and Western blotting, it was shown that the tumour suppressor, p53, upregulates genes involved in cell‐cycle arrest and apoptosis including p21 and *PUMA*, respectively (Figure 5). A balance between p21 and *PUMA* has been recently known in response to exogenous p53 expression in human colorectal cancer cells.^33^

Moreover, ROS assay showed that 6Sh may generate reactive oxygen species in the leukaemic cell line (Figure 6). It was previously declared that ROS may cause DNA damage, contributing to p53 activation followed by apoptosis.^34^, ^35^ Interestingly, *FASN* overexpression is recently introduced by our group as a negative prognostic factor for paediatric ALL.^24^ Additionally, the association of FASN with resistance to chemotherapeutic drugs was previously determined.^36^ Supportingly, published data revealed that FASN inhibitors may block proliferation and induce apoptosis in CML cell lines.^37^ Considering the identified side effects of chemical FASN inhibitors, scientists prefer to use the low cost and abundant natural herbal compounds instead, which may block the FASN activity in order to conquer cancer.^38^ The precise signalling pathway regulating FASN expression in different cancers is yet to be established. However, according to the Impheng and colleagues’ results, increasing ROS levels may suppress FASN expression through dysregulation of several intracellular signalling pathways including PI3K/AKT, mTOR and MAPK.^39^ Considering our data, 6Sh may induce p53 activation in leukaemic cells, leading to apoptosis and cell‐cycle arrest. Furthermore, 6Sh may generate high cytoplasmic levels of ROS in ALL malignant cells, contributing to the downregulation of *FASN* and cell death. These data are novel findings which may shed light to a possible way for overcoming drug resistance in paediatric ALL. However, mechanistic studies are required to confirm the causative impact of 6Sh on *FASN*.

**FIGURE 7:**
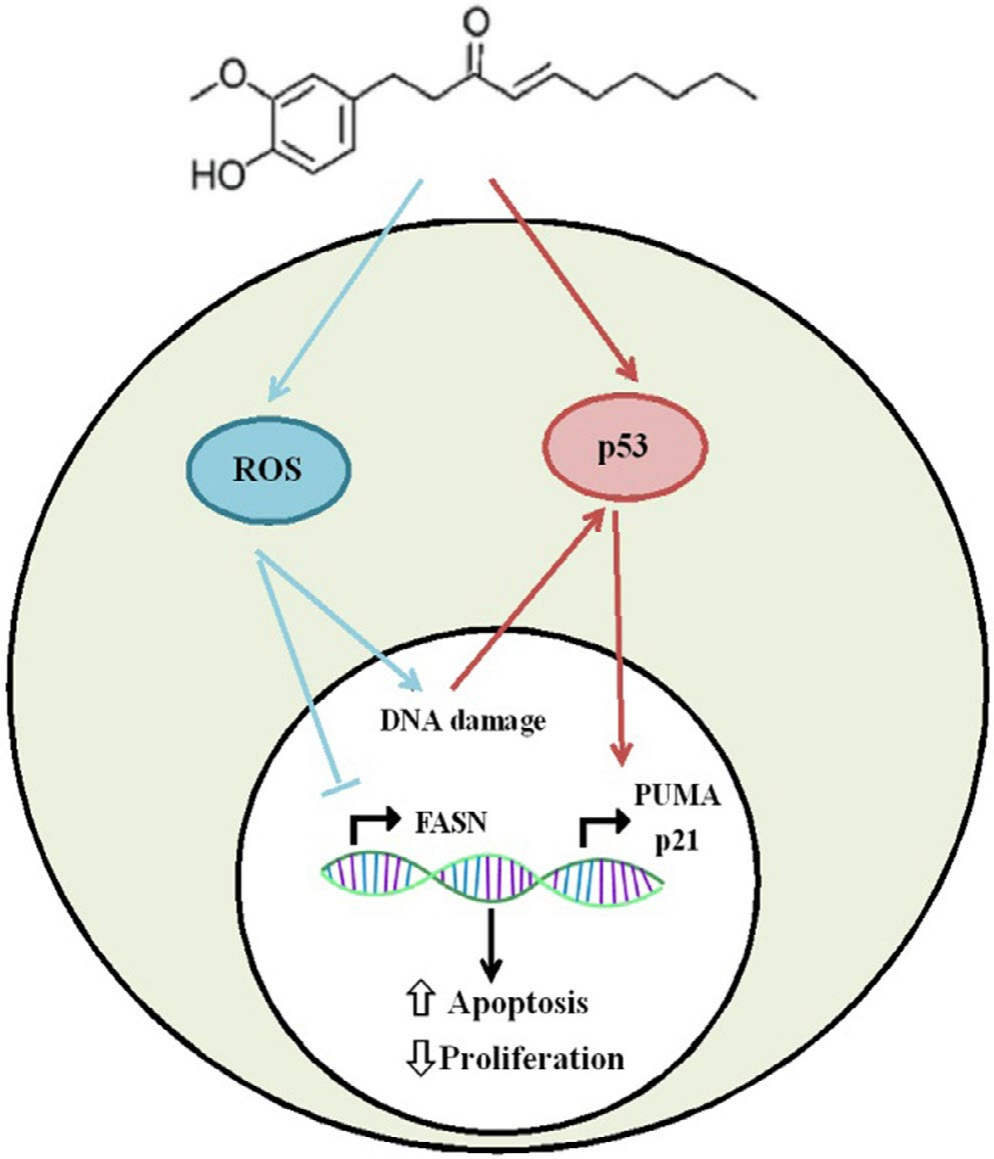
Six‐shogaol, a small molecule with anti‐proliferative and apoptotic effects. The schematic diagram demonstrates major pathways by which 6Sh may confer anti‐leukemic effects. 6‐Shogaol may, simultaneously, upregulate the p53 expression levels and generate reactive oxygen species (ROS). Consequently, ROS may inhibit fatty acid synthase (*FASN*) expression and cause DNA damage. FASN suppression, in its turn, induces apoptosis and decreases cell growth. Additionally, DNA damage leads to the activation of p53, which, as a transcription factor, upregulates the expression levels of the apoptotic genes and those involved in the cell‐cycle arrest, including *PUMA* and *p21,* respectively. 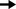 = activation, 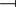 = inhibition, 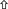 = induction, 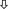 = suppression, 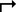 = gene expression

Taken together, the current study is the first research demonstrating the anti‐cancer effect of ginger extract on ALL mice models. Moreover, the specific anti‐leukaemic derivative of this pharmaceutical medicine is identified. 6‐Shogaol is introduced as a novel naturally occurring small molecule, which may selectively and synergistically amplify the cytotoxicity of MTX on the malignant lymphoblasts. 6Sh may exert its anti‐neoplastic effects through activation of p53 and generation of ROS, leading to apoptosis and cell‐cycle arrest, or downregulation of fatty acid synthesis, respectively (Figure 7). Since cancer cells often use specific signalling pathways for growth and proliferation, identifying 6‐shogaol as the most effective derivative of ginger and illuminating its molecular anti‐leukaemic mechanism can be an important step in targeted therapy.

## CONFLICT OF INTERESTS

The authors declare that they have no competing interests.

## AUTHOR CONTRIBUTION

**Somayeh Najafi Dorcheh:** Data curation (lead); Formal analysis (lead); Investigation (lead); Visualization (lead); Writing‐original draft (equal). **Soheila Rahgozar:** Conceptualization (lead); Funding acquisition (equal); Methodology (lead); Project administration (lead); Resources (lead); Supervision (lead); Validation (lead); Writing‐original draft (equal); Writing‐review & editing (lead). **Daryush Talei:** Funding acquisition (equal); Project administration (supporting); Supervision (supporting).

## ETHICAL APPROVAL

All the patients’ parents were informed about the purposes of the study and consequently have signed their “consent of the patient”. All investigations conformed to the principles outlined in the Declaration of Helsinki and were performed with permission by the responsible Ethics Committee of the University of Isfahan (agreement number IR.UI.REC.1398.009 for human studies, and ethics number IR.UI.REC.1396.056 for animal studies).

## Data Availability

All data generated or analysed during the current study are included in this article. Supplementary and raw data are available if required.
